# The effect of self-perceived stress, the history of smoking and drinking on weight status in Chinese adults - evidence from the 2015 China Health and Nutrition Survey: A cross-sectional study in Chongqing, China

**DOI:** 10.1097/MD.0000000000021159

**Published:** 2020-07-31

**Authors:** Fang Yuan, Mengyun Wu, Wei Li, Huadong Zhang

**Affiliations:** Department of Occupational Health and Radiological Health, Chongqing Center for Disease Control and prevention, Chongqing, 400042, China.

**Keywords:** drinking, self-perceived stress, smoking, weight status

## Abstract

Being overweight and obese is a serious public health issues in China. However, the effects of substance use and mental factors on weight changes remain unclear. In this study, we aimed to investigate the association among self-perceived stress, history of smoking and drinking, and weight status by using data from the China Health and Nutrition Survey in 2015.

A total of 8028 adults were selected from China Health and Nutrition Survey in 2015. The self-reported data primarily included sociodemographic data, self-perceived stress scores, and history of smoking and drinking. Physical measurements including height and weight were logged to calculate body mass index. Multivariate and multinomial regression models were used to estimate effects of substance and perceived stress on weight status.

The prevalence of underweight and overweight/obese people were 4.52% and 51.51% in Chinese adults, respectively. Adults with high perceived stress were negatively associated with being overweight and obese (OR = 0.80, 95% CI = 0.66–0.97 in the middle level and OR = 0.69, 95%CI = 0.55–0.88 in the high level). Adults with history of smoking had low risk of being overweight/obese (OR = 0.71, 95% CI = 0.62–0.82). Adults with history of drinking had high risk of overweight/obese (OR = 1.22, 95% CI = 1.06–1.40). In addition, the association between drinking and overweight/obese was affected by different levels of perceived stress (OR = 1.15, 95%CI = 0.83–1.59 in low-stress group and OR = 1.42, 95%CI = 1.04–1.94 in high-stress group).

The effects of self-perceived stress and history of smoking as well as drinking on weight status were significant in this study. The government and healthcare policymakers should strengthen early psychological factor and behavioral intervention to decrease the prevalence of abnormal-weight status.

## 1 Introduction

With the widespread studies on nutrition, overweight and obesity are serious public health concerns. The National Health Commission of the People's Republic of China showed that the prevalence of overweight and obesity are 30.1% and 11.9% in adults and 9.6% and 6.4% in adolescents, respectively.^[1]^ The number of individuals with abnormal weight is still increasing because of the large population of China. Overweight/obesity is associated with many chronic diseases, such as hypertension, cardiovascular disease, diabetes, and cancer.^[2–4]^

Meanwhile, individual stressful status also attracted the attention of researchers. Self-perceived stress is the individual ability to deal with risk behavior and emotional problems, and vulnerable people are sensitive to stress threshold. By contrast, low perceived stress was beneficial for people's health because people with low stress was associated with positive social support, which can play the “buffering” role of negative emotions.^[5]^

Previous studies reported the association of stress with health status and behavior. For example, work stress is associated with hypertension, peptic ulcer, and cancer.^[6]^ Moreover, perceived stress is at an increased risk of substance dependence. A large cross-sectional survey from 41 countries showed that increased stress is associated with smoking in Africa, America, and Asia.^[7]^ However, the relationship of perceived stress with alcohol behavior is controversial and unclear. In a clinical trial, patients with high anxiety level are strongly associated with alcohol use disorder; this association is not present in perceived stress.^[8]^ Empirical research also suggested that high stress is associated with cigarette smoking, recent increase in smoking, and low self-efficacy to quit smoking in working adults, but work stress does not predict alcohol use.^[9]^ A meta-analysis from Europe indicated that nondrinkers and heavy drinkers are connected with job strain compared with moderate drinkers in cross-sectional and longitudinal studies.^[10]^ Hence, stress and substance can pose combined effects on individual health.

The association between mental condition and weight status has been widely studied and discussed. Individual psychological factors can influence weight level through the change in substance use. A cross-sectional in China found that current depression accounts for higher risk than smoking in obese women, but this result is insignificant in underweight/normal and overweight groups.^[11]^ A longitudinal study reported that body mass index (BMI) score were significantly higher in the moderate- and higher-stress groups than the lower-stress group during the all 5-year study..^[12]^ One possible explanation for this phenomenon is that substance plays potential role between negative emotion and weight status, that is, negative emotion would increase the frequency of substance use to impose indirect effect on weight.^[13]^

However, the role of stress is few between substance and obese in previous studies, and stress may have special mechanism in this relationship. For example, teenagers are more vulnerable than adults in terms of difficulty; workers have higher stress tolerance than nonworkers. The different stress levels would be extremely significant in various groups. Evaluating the degrees of stresses would help in understanding its moderating role in various group. A literature revealed that although this difference is unsupported between nonsmokers and smokers in terms of BMI and perceived stress, nonsmokers with high perceive stress level reported increased food intake, and smokers reported eating less than usual.^[14]^ A empirical research showed that both the perceived stress and all alcohol consumption habits with respect to obesity had significant interactions.^[15]^ In summary, the effects of perceive stress on weight change should be studied by different levels.

The majority of studies primarily considers the association between the mental condition and substance use among adolescent. Few studies have examined the moderating effect of perceive stress between substance use and weight changes by using a large sample. To understand this mechanism in Chinese environment. We collected the data from China Health and Nutrition Survey (CHNS) in 2015 to investigate the main effect of self-perceived stress and the history of drinking/smoking on weight status in Chinese adults. Meanwhile, we also explored the moderating effect of self-perceived stress between the history of drinking/smoking and weight status among Chinese adults.

## 2 Materials and methods

### 2.1 Ethical review

The CHNS has been approved by institutional review boards at the University of North Carolina at Chapel Hill and the National Institute for Nutrition and Food Safety, China Centre for Disease Control and Prevention. All participants provided written informed consent for their participation in the survey.

### 2.2 Data

The study data were derived from CHNS, which is an ongoing longitudinal survey project that was established and conducted by Chinese Center for Disease Control and Prevention and the University of North Carolina at Chapel Hill. These panel data were used to investigate the change in Chinese economic and social transformation with regard to its influence on the health and nutrition of Chinese people and assess the Chinese government's policies on how it affects medical resources allocated since 1989. Follow-up data were updated in 1991, 1993, 1997, 2000, 2004, 2006, 2011, and 2015. This study included most of Chinese provinces, including 12 provinces and 3 municipalities (e.g., Chongqing, Shanghai, and Beijing). The 2015 wave data was used in this study, and available website was supported to download questionnaires and data (https://www.cpc.unc.edu/projects/china).

### 2.3 Study design and sample

CHNS adopted a multistage and random cluster sampling in urban and rural areas. Reference to regional economic level (eg, low, middle, and high), cities and counties per province were stratified. Then, 2 urban and 2 suburban areas within cities, 1 community in down, and 3 villages within counties were randomly selected. A total of 13 890 adults and 5584 households were enrolled in the 2015 dataset. In present study, we primarily chose the individual survey of demographic data and health services data. In particular, CHNS first designed the scale of self-perceived stress to explore adults’ mental health in 2015. Enrolled participants who missed research and control variables were excluded. Finally, 8028 subjects who aged above 18 were included into current analysis in total.

### 2.4 Self-perceived stress

The independent variable was the individual self-perceived stress scores, which was calculated using 14 items. The participants rated each item on a 5-point scale, which ranged from 1 (never) to 5 (always); items 4 to 6, 9, 10, and 13 were reversely scored. High scores indicated high perceived stress possibility, as shown in Table 1. This scale demonstrated high reliability (Cronbach's α = 0.81). Then, perceived stress was divided into 3 levels with regard to its z-scores. Low tier was defined as below mean subtracting 1 SD, middle tier was considered between mean±1 SD, and high tier was defined as above mean adding 1 SD. The questionnaire was as follows:

**Table 1:**
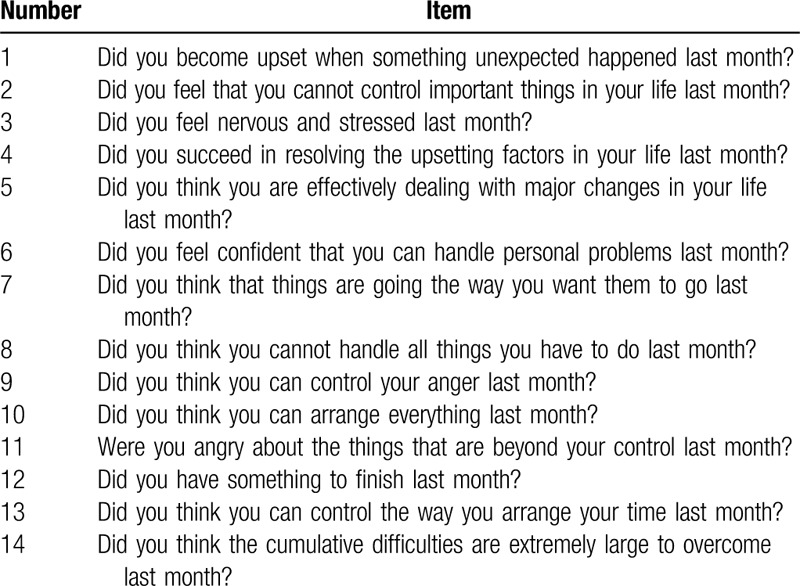
The questionnaire of self-perceived stress (n = 8028).

### 2.5 BMI and weight status

The dependent variables were BMI and weight status, which were derived through 2 steps. First, BMI was calculated as weight (kg)/height (m^2^). Second, the weight status was categorized into 4 types,^[16]^ namely, underweight (BMI < 18.5 kg/m^2^), normal weight (18.5 kg/m^2^ ≤ BMI < 24 kg/m^2^), overweight (24 kg/m^2^ ≤ BMI < 28 kg/m^2^), and obese (≥28 kg/m^2^).

### 2.6 History of alcohol and tobacco consumption

Alcohol and tobacco use may be important predictor for the changes in weight status. In the CHNS database, the history of alcohol and tobacco use were logged by 2 questions, as follows:

(1) “have you drunk beer, liquor, or other alcoholic beverages in 2014?” (yes or no) and
(2) “Do you smoke (including manual cigarettes, mechanical cigarettes, and pipe)?” (yes or no).

### 2.7 Control variables

Control variables involved individual and household's sociodemographic variables, such as gender, age, residence, highest education levels, working status, marital status, living with parents, and tobacco consumption. Education was divided into junior high school and below, high school, and college and above. Marital status was divided into single, married, and separated/divorced/widowed. Residence was divided into urban and rural. Household income was split by percentage (eg, Q1, Q2, Q3, and Q4).

### 2.8 Statistical analysis

The continuous data were described using mean ± SD, and the categories of data were proportions (%). Chi-squared and ANOVA tests were adopted to examine the difference between weight status and related variables. First, nested multivariate linear regression was used to assess the association among predictors and BMI value. Three models were used, as follows: model 1 adjusted for residence and gender; model 2 was further adjusted for perceived stress level; model 3 was further adjusted for other sociodemographic data and history of tobacco, as well as alcohol consumption, in 2014. We also utilized nested multinomial logistic regression to explore the effect of independent variables on unhealthy status (eg, underweight and overweight/obese). All the analyses were performed using STATA 15.1 (Stata Corporation, College Station, TX). Statistical significance was considered when *P* < .05 (2-sided).

## 3 Results

### 3.1 Sample characteristics of participants

**Figure 1:**
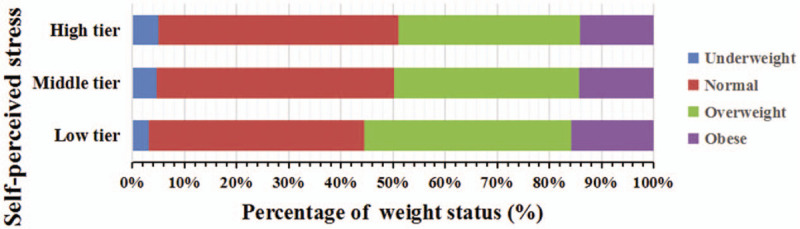
Adults’ weight status distribution of different self-perceived stress levels.

A total of 8028 people was included in this study, of which 4.52% were underweight, and 51.51% were overweight/obese. Suburban or rural village has higher proportion of malnutrition than urban ones (40.22% of underweight in urban and 59.78% of underweight in rural; 47.10% of obese in urban and 52.90% of obese in rural). The average self-perceived stress scores were 33.52 ± 6.69. In subgroup analysis, the difference was significant between the degree of stress and weight status (*P* < .001). The distribution of weight status showed certain differences between males and females (*P* < .001), that is, 59.23% accounted for underweight females, and 51.50% accounted for overweight/obese males. In 2015, 26.17% of adults reported that they had history of tobacco consumption in the underweight group, and 26.38% of the participants reported this rate in the overweight/obese group. A total of 20.04% of adults reported history of drinking in the underweight group in 2014, while this rate significantly increased to 30.46% in the overweight/obese group (Table 2). The bar graph results showed that the proportion of overweight/obese people was larger than those with normal weight in the low tier of perceive stress, while this phenomenon was opposite in high tier (Fig. 1).

**Table 2:**
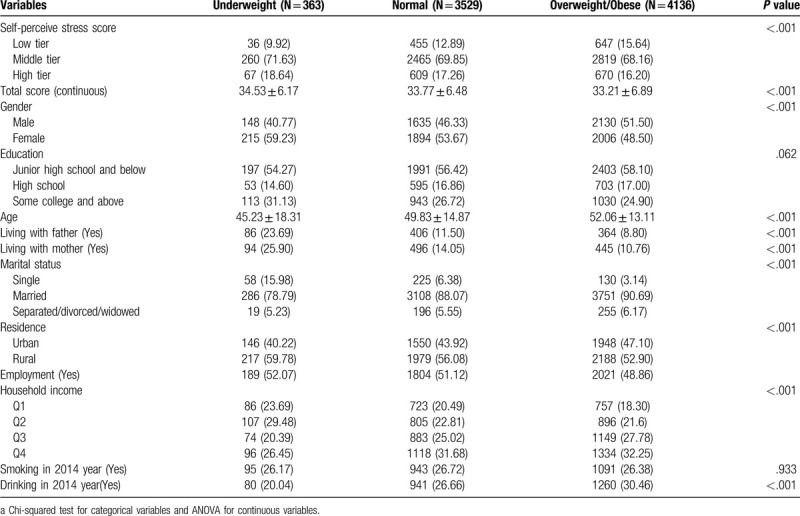
Sample characteristics of survey by gender in 2015 (N = 8028).

### 3.2 Multivariate regression model analysis for BMI values among Chinese adults

The multivariate regression model indicated that BMI values showed significant difference in gender and residence in model 1. Adults living in urban were more likely to be high weight, and the weight of males was 65% higher than that of females (*P* < .01). In model 2, after entering perceived stress, the association between BMI and perceived stress was negative (*P* < .01). In model 3, we conducted a full model with history of smoking and drinking. The results indicated that the effect of perceived stress was still significant after controlling the full covariates (*P* < .001). In addition, people with history of smoking are also an important predictor of BMI for adults in low BMI value, whereas history of drinking showed positive effects on BMI values. College education may play negative roles in the increase in BMI (*P* < .05). Lastly, marriage history and household income belonging to the middle level can also predict an increasing weight (*P* < .001), as shown in Table 3.

**Table 3:**
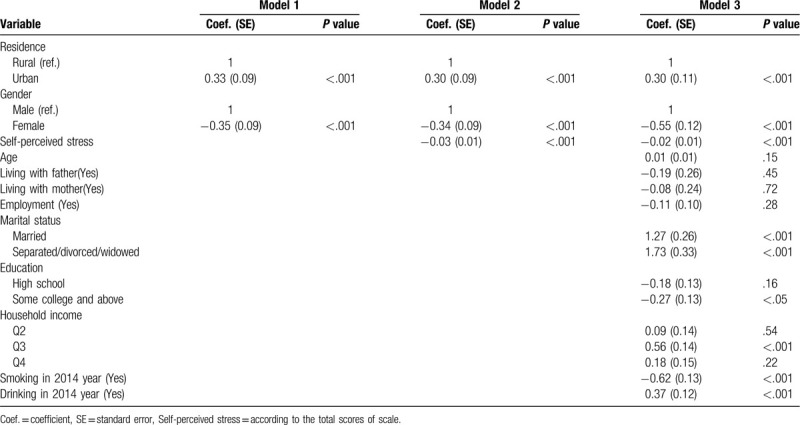
Effects of self-perceived stress and tobacco and alcohol use on BMI values among adults.

### 3.3 Multinomial logistic regression analysis for BMI values among Chinese adults

To understand the association among psychological factor, substance use, and different weight statuses, we conducted multinomial logistic regression by using 3 models. In model 1, we only included residence and gender into model, and the association in overweight/obese subjects was significant. In model 2, we included perceived stress as ordered variables into the model, thereby indicating people with middle and high stress levels had less risks of being overweight/obese than those with low level stress (OR = 0.81, 95% CI = 0.71–0.93 in middle stress level and OR = 0.78, 95% CI = 0.67 to 0.92 in high stress level), but this association was not found between the underweight and normal groups. In model 3, we included other confounding factors, such as the history of alcohol and tobacco. The association between perceived stress and overweight/obese was still significant (OR = 0.80, 95%CI = 0.66–0.97 in middle stress level; OR = 0.69, 95%CI = 0.55–0.88 in high stress level). Moreover, people with history of smoking had lower risks of being overweight or obese than nonsmoker (OR = 0.71, 95%CI = 0.62 to 0.82). The adults with drinking were more likely to become overweight and obese than nondrinkers (OR = 1.22, 95% CI = 1.06 to 1.40), and underweight and history of drinking was unassociated. Residence, gender, age, marital status, education, and household income were also associated with weight status, as shown in Table 4.

**Table 4:**
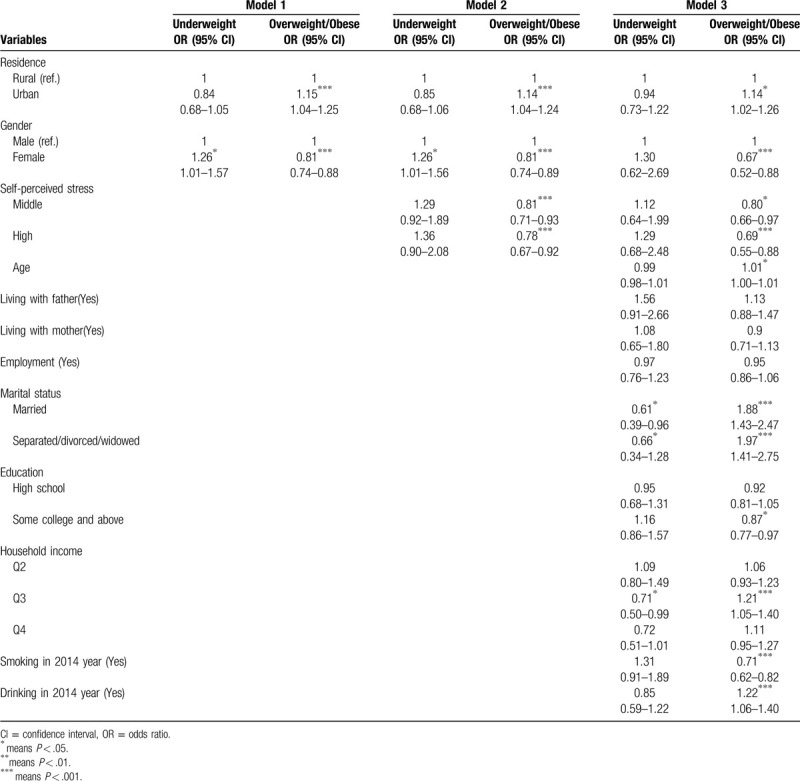
Effects of self-perceived stress and history of smoking and drinking on weight status among adults.

### 3.4 Multinomial Logistic regression for weight status by perceived stress

We conducted subgroup to analyses the change of substance’ effect on weight status. We did still not find the significant effect of substance on underweight models with different perceived stress. In addition, perceived stress cannot moderate the effect of smoking on overweight/obese in full stressful level (Range: OR = 0.67–0.77, 95%CI = 0.47–0.95). However, the moderation of perceived stress was examined between drinking and overweight/obese. In model 1 and model 2, the association between drinking and overweight/obese was insignificant. In model 3, drinker had great odds of overweight/obese (OR = 1.42, 95%CI = 1.04–1.94). This result indicated that drinking was a important predictor of overweight/obese in high-level perceived stress, not for low-level perceived stress. (See Table 5).

**Table 5:**
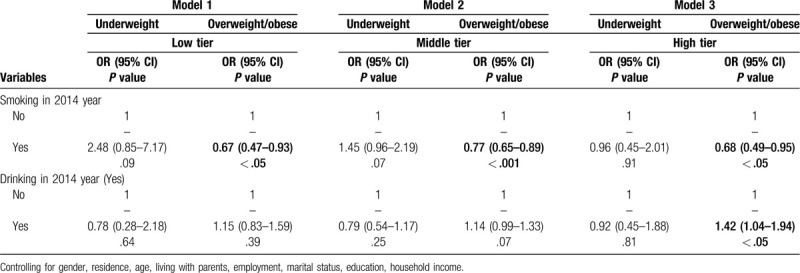
Mutinomial logistic regression for weight status by perceived stress.

## 4 Discussion

The strength of relationship among mental factor, substance use, and weight status is not always homogeneous because of the demographic difference in the country.^[11–13]^ The prevalence of overweight and obese people in the present study were 35.95% and 15.56%, which were higher than the national level in 2015.^[1]^ This rate also was higher than those observed previously in China.^[17–19]^ In the subgroup analysis, the percentages of males were significantly higher than those of females in the overweight/obese group (male: 51.50% and female: 48.50%), and females were a considerable majority in the underweight group compared with males (female: 59.23% and male: 40.77%). According to previous national monitoring data in 2010, the prevalence of overweight was 31.5% in men and 29.7% in women; the proportion of obese was 11.9% in men and 12.1% in women.^[18]^ The present study indicated that overweight and obesity of adult males had become a serious issue, which should draw the attention of the government and healthcare facilities.

The present study found that perceived stress was negative associated with adult's weight status. Continues stress can regulate and affect fat storage, as well as change the microbial colonies in host, thereby leading to the vicious circle of overeating.^[20–22]^ Moreover, a longitudinal study found that perceived stress is positively associated with leisure time and physical activity, as well as frequent fast food consumption.^[21]^ Another cross-sectional study pointed out that transit workers have high BMI and poor dietary habit when working in the highest work hours.^[23]^ However, our results were inconsistent with those of previous studies, and both associations were negative. This result can be explained in several manner. First, people with high stress would increase the perception of body image, thereby seeking slender shape, especially in younger adults.^[24]^ Second, the evidence from low- and middle-income countries should be supplemented. Developing countries were popular with vegetarian dietary model, that is, accounting for large dietary and cereals in daily diet compared with Western dietary pattern, and the decline in high fat food consumption can also influence the association between stressful diet and changes in weight.^[25]^ Third, the consumptions of tobacco and alcohol were typical among adults in China. Adults can experience individual stress by substance use increase of stressful diet.^[26,27]^

The risk of overweight or obese adults that were smoking was 29% lower than that of nonsmoker. This result indicated that smoking was negatively associated with BMI with the increase in emaciation rate and decrease in obese rate. The mechanism of smoking on weight management was unclear. One evidence suggested that carbon monoxide and nicotine in smoke can affect the digestive system through the jury of hypoxia on the nervous system with chronic gastritis and gastric ulcer.^[28]^ Smoking can also inhibit pancreatic exocrine secretion, thereby influencing the amount of food and even triggering pancreatic cancer.^[29]^ Smoking can excite sympathetic nerves, thereby increasing energy consumption. Even former smoker had an average of 4–5 kg compared with current smoker.^[30]^ However, smoking is associated with massive chronic disease, such as the association with hypertension and diabetes, which bring harmful outcome more than benefit of weight decreased. In China, smoking is a popular factor in low- and middle-income areas; these people have high risks of being malnourished and to lack healthcare facilities.^[31,32]^ Important environment and culture factors should be studied in the future. A total of 59.78% of underweight were from rural areas in CHNS 2015, which can provide a new sight into the relationship of tobacco use and overweight/obesity in urban–rural disparity.

The history of alcohol consumption showed contrasting results. People with history of alcohol have prevailing sedentary behavior.^[33]^ One reason for this result is the clustering of unhealthy lifestyle behavior, such as smoking, overnutrition, and physical inactivity, in this group.^[34,35]^ These factors increase the risk of being overweight and obese. Although people with history of drinking are associated with physical activities, they also have increased frequency of unhealthy dietary behavior, such as eating barbecue and fast food.^[36,37]^ These results indicated that the association between weight status and alcohol consumption had a complex connection.

Adults with college degree have low risk of being overweight/obese. Moore and Cunningham^[38]^ pointed out that individual with high social status tends to have low stress levels and healthy weight status. Adults with high degree have received scientific knowledge and obtained high social position, while this group can intend to keep healthy and perform exercise compared with those with low education level.^[39]^ Moreover, middle income level was a significant predictor for overweight and obese. Similar to high education, rich men can develop healthy lifestyle behavior and keep their body weight in the normal level. Middle- and low-income levels have increased risks of poor nutrition because of fluctuating income and low education. Moreover, people with history of marriage are likely to be overweight and obese. Single adults have higher demands for body image to seek spouse. Hence, this group can keep their weight in the normal level. However, married adults should exert effort to work hard, which can influence one's weight. Marital strain and dissatisfaction can increase the risk of body weight gained because of the interfered self-regulatory behavior.^[40]^

The present study has several limitations. First, this study lacks panel data on individual's mental factors and body weight. Therefore, we cannot derive the causal effect in this cross-sectional study. Second, although we controlled several confounding variables into this model, the physical activity, sedentary behavior, and nutrition data are missing, and these materials can be extremely important in exploring the change in weight status. Third, the CHNS data showed nested structure among provinces, communities, and individuals. Therefore, the effect of cross-level should be considered in analysis, and mix effect model may lose large information, thereby decreasing test efficiency. Fourth, information of chronic disease is limitation in 2015 CHNS with mass missing and selected by self-reported versions. Hence, we cannot assess the influence of chronic disease on weight. This may weaken the validity of the study.

## 5 Conclusions

The prevalence of overweight and obese individuals was relatively high among Chinese adults in 2015. The study suggested that high perceived stress level may cover the risk of being overweight and obese among Chinese adults, but not for predicting underweight status. People with history of smoking had low risk to be overweight and obese. Alcohol consumption was likely to increase abnormal weight. Early psychological and behavior intervention should be implemented to prevent and improve Chinese adults’ weight status.

## Acknowledgments

The authors express their thanks to National Institute for Nutrition and Health, China Centre for Disease Control and Prevention and the University of North Carolina at Chaple Hill, NIH in CHNS project's contribution. We also grateful to Manoj Sharma of Jackson State University for assistance in manuscript language polishing and advices.

## Author contributions

Conceptualization, H.Y and MY.W; Methodology, W.L. and Y.Z; Validation, HD.Z; Formal Analysis, H.Y; Data Curation, MY.W; Writing-Original Draft Preparation, H.Y and MY.W; Writing-Review & Editing, HD.Z; Visualization, H.Y. and MY.W.
